# Sentinel lymph node detection for lung cancer surgery: a possible pathological surrogate of overall lymph node dissection

**DOI:** 10.3389/fonc.2025.1474887

**Published:** 2025-03-14

**Authors:** Florent Stasiak, Joseph Seitlinger, Lyndon C. Walsh, Arthur Streit, Joelle Siat, Guillaume Gauchotte, Lucie Schnedecker, Stéphane Renaud

**Affiliations:** ^1^ Department of Thoracic Surgery, Nancy Regional University Hospital, Nancy, France; ^2^ Department of Pathology and Molecular Biology, Nancy Regional University Hospital, Nancy, France; ^3^ Research Unit Institut National de la Santé Et de la Recherche Médicale (INSERM) U1256, Nutrition-Génétique et Exposition aux Risques Environnementaux (NGERE) Unit, Lorraine University, Nancy, France

**Keywords:** sentinel lymph node, lung cancer, thoracic surgery, lymph node dissection, indocyanin green (ICG)

## Abstract

**Background:**

Systematic lymph node dissection (SLND) is currently the gold standard for lung cancer surgery. However, this is not the case for breast cancer or melanoma, where sentinel lymph node (SLN) identification is routine. The SLN could be a possible surrogate for the pathological status of the other lymph nodes, but there is limited data in the literature for lung cancer surgery. The main objective of this study was to evaluate pathological concordance between the SLN and the complete lymphadenectomy.

**Methods:**

In this retrospective study, we reviewed all cases of localized lung cancer that had benefited from our SLN identification protocol and underwent surgery (segmentectomy or lobectomy) between December 2020 and December 2023. We examined the pathological status of the SLN and the rest of the lymph node dissection to assess the pathological concordance rate.

**Results:**

After exclusion, 106 patients with localized stage I-IIA non-small cell lung cancer and suspected node negative disease (N0) were included in our study. Of these 106 patients, 96 had a pN0 SLN (90.6%) and 10 had a positive SLN (pN+), resulting in an upstaging rate of 9.4%. All patients with a pN0 SLN were also pN0 for the rest of the lymph node dissection, corresponding to a pathological concordance rate of 100%. Disease-free survival was statistically lower in the pN+ SLN group than in the pN0 SLN group (p<0.0001).

**Conclusion:**

We demonstrated a 100% pathological concordance between SLN when it is cancer-free and the rest of the lymph nodes in the lymph node dissection, suggesting that the SLN is a good indicator of the overall pathological status of the other lymph nodes in the thorax.

## 1 Introduction

Lung cancer remains the leading cause of cancer-related mortality worldwide (1). It is well known that the pulmonary or mediastinal lymphatic system represents a common pathway for metastatic dissemination of lung cancer (2). According to the recommendations of both European Society of Thoracic Surgeons (ESTS) of 2006 and French Society of Thoracic and Cardiovascular Surgeons (SFCTCV) of 2008, systematic lymph node dissection (SLND) remains the gold standard of care in lung cancer surgery. Particularly, the monobloc excision of at least 3 hilar and interlobar lymph node chains and 3 different mediastinal chains, including the subcarinal chain (3). This SLND enables the best assessment of the pathological nodal involvement of the disease (4).

However, SLND is not the standard treatment for all cancers. The sentinel lymph node (SLN) technique was first described in 1992 and is now routinely used in the treatment of melanoma and breast cancer, for example (5, 6). The SLN, by definition, corresponds to the first lymph node relay, receiving lymphatic afferents from a drainage zone and, and unlike other lymph nodes in the lymph node dissection (LND), is analyzed completely, with finer sections and time-consuming dedicated immune-histochemistry techniques, allowing for increased detection rates of micro-metastases (7). The importance of identifying a SLN relies on the assessment of the potential for dissemination to distant sites. Patients with early-stage cN0 NSCLC have a 70% 5‐year survival rate, suggesting that many cN0 patients have lymph node metastasis (8). There is a clear need for accurate prediction of nodal disease, which will aid in the design of new therapeutic strategies. Since 1999, attempts have been made to adapt the SLN technique for lung cancer, by using various markers (9). The use of methylene blue has failed to achieve more than 50% identification of the SLN, while the use of radiotracers has shown highly variable results, ranging from 50% to 80% identification, with significant complications and organizational difficulties, particularly with nuclear medicine (10–12). More recently, the use of indocyanine green (ICG) and near-infrared (NIR) fluorescence imaging has given renewed interest to the sentinel node in thoracic surgery. Several teams, including ours, have already demonstrated safety and feasibility of this technique, with a SLN identification rate close to 80% without any adverse events associated with the use of ICG (13–16).

The SLN technique has become a standard of care in cT1-T2N0 breast cancer (17), even though false negatives can be as high as 30% (18). There are currently limited data in the literature concerning the pathological concordance between the SLN and the rest of the LND and existence of false negatives in lung cancer.

We therefore conducted a study with the primary objective of assessing the pathological concordance between the SLN and the rest of the LND. Our primary outcome was to assess the pathological concordance between the SLN and the rest of the lymph nodes in the complementary LND. The secondary aim was to assess the disease-free survival (DFS) and overall survival (OS) between SLN+ and SLN- cohorts.

## 2 Materials and methods

This retrospective, observational, monocentric study was carried out in the Thoracic Surgery Department of the Centre Hospitalier Régional Universitaire de Nancy (France). Written consent to participate in the study was obtained from all patients included.

### 2.1 Study population & inclusion criteria

Adult patients (≥ 18 years old) with proven or suspected surgically resectable cT1a-cT2b N0 (clinical stage IA to IIA) non-small cell lung cancer (NSCLC), without suspicion of lymph node involvement on preoperative 18F-Fluoro-deoxy-D-glucose positron emission tomography (18F-FDG PET), and who had given written consent, were included in this study between December 2020 and December 2023. Each patient’s preoperative thoracic computed tomography (CT) scan, 18F-FDG PET scan and brain MRI were interpreted by a radiologist and a nuclear radiologist specialized in thoracic oncology. Assessment of pulmonary function tests, as well as staging, were systematically performed according to European Respiratory Society (ERS)/ESTS recommendations (19). Pathological stage was determined according to the eighth edition of the TNM lung cancer staging system (20).

### 2.2 Sentinel node marking & evaluation technique

Injection of ICG was performed as previously described by our team (14). In summary, a dilution of 1mL of ICG in 20% human albumin is injected into the tumor region in all patients. Injection was preferentially performed by electromagnetic navigational bronchoscopy (ENB), or by a direct transpleural injection approach.

In cases of the direct transpleural approach, ICG was injected through the incision by a 19G fine needle (Arcpoint ^®^, Medtronic, Minneapolis, MN, USA) into the peritumoral area at a depth of at least 1 cm in the parenchyma to limit diffusion of ICG in the chest cavity.

Navigation in the airways was performed by ENB using the Illumisite ^®^ platform from Medtronic (Minneapolis, MN, USA). Once near the lesion, a 19G needle (Arcpoint ^®^, Medtronic, Minneapolis, USA) was inserted through the catheter, and injection of ICG was performed.

**Figure 1:**
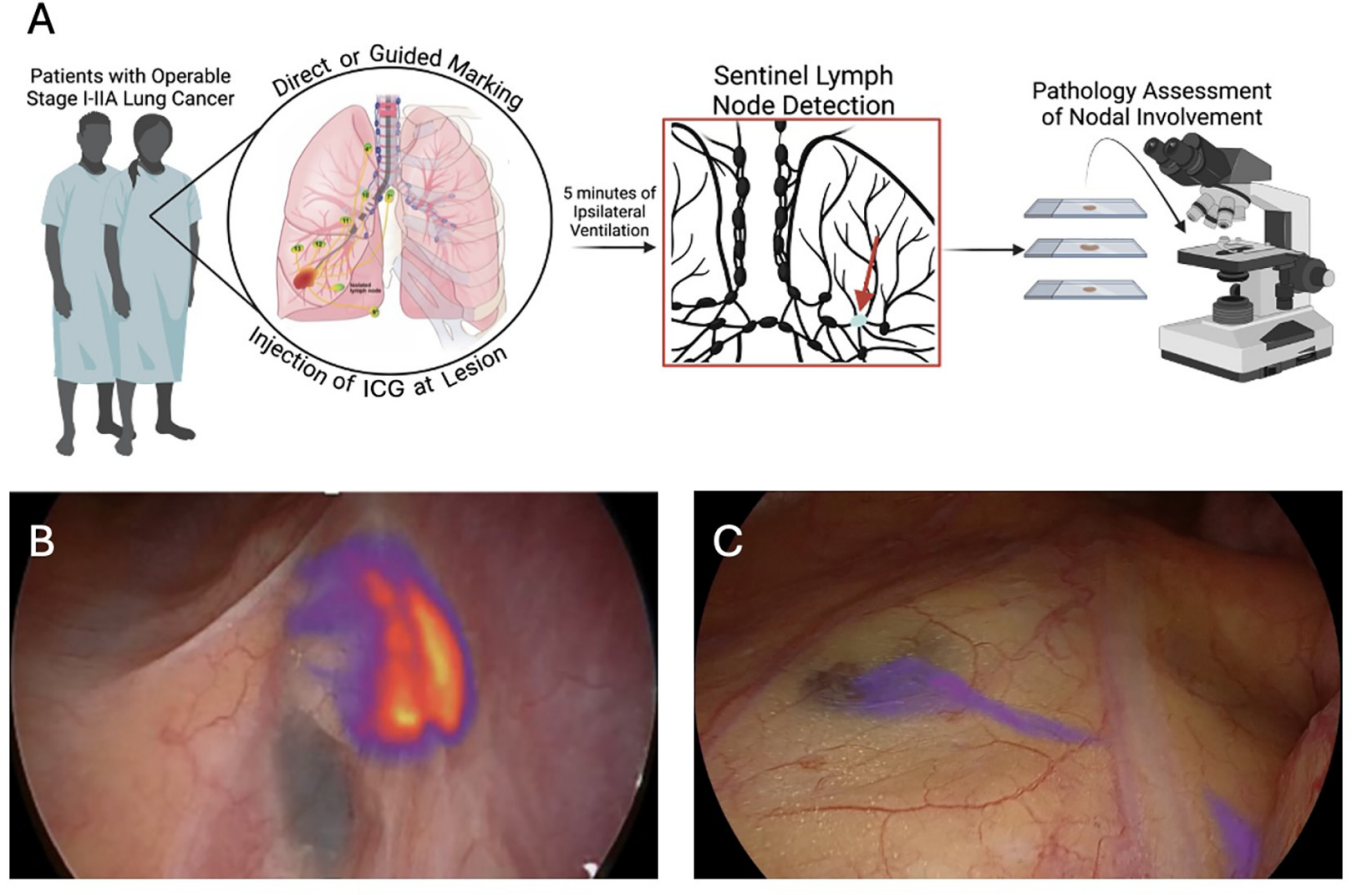
Sentinel node detection protocol. **(A)** General flow of sentinel node protocol; **(B)** Sentinel node detection in station 7; **(C)** Sentinel node in station 3a with a visualized lymphatic pathway from the left upper lobe.

The detection of SLN by VISIOSENSE^®^ (Medtronic) infrared camera was initiated after at least 5 minutes of ipsilateral ventilation. If a node was found to be fluorescent, it was resected, followed by SLND (stations 2, 4, 7, 8, 9, 10, 11, 12 for the right-hand side, stations 2, 5, 7, 9, 10, 11, 12 for the left-hand side), as recommended by ESTS guidelines (3). Once resected, the SLN was sent to the pathology department apart from the other lymph nodes, where it was analyzed *in toto* and via immuno-histochemistry with anti-cytokeratin AE1/AE3 antibodies. The non-SLN lymph nodes were analyzed using a standard technique, with hematoxylin-eosin staining An illustration is provided in **Figure 1**.

Segmentectomy was performed for peripheral lesions of less than 2cm, cN0, or in patients with poor functional reserve who could not tolerate lobectomy, as recommended (21).

### 2.3 Statistical analyses

Descriptive statistical analyses were conducted to evaluate the general characteristics and frequency of variables. Log-rank tests and hazard ratios were used to assess the DFS of the patient cohort. All statistical tests and visualizations were conducted using Prism by GraphPad v.10.2.2 (Boston, USA) and BioRender.com.

## 3 Results

**Figure 2:**
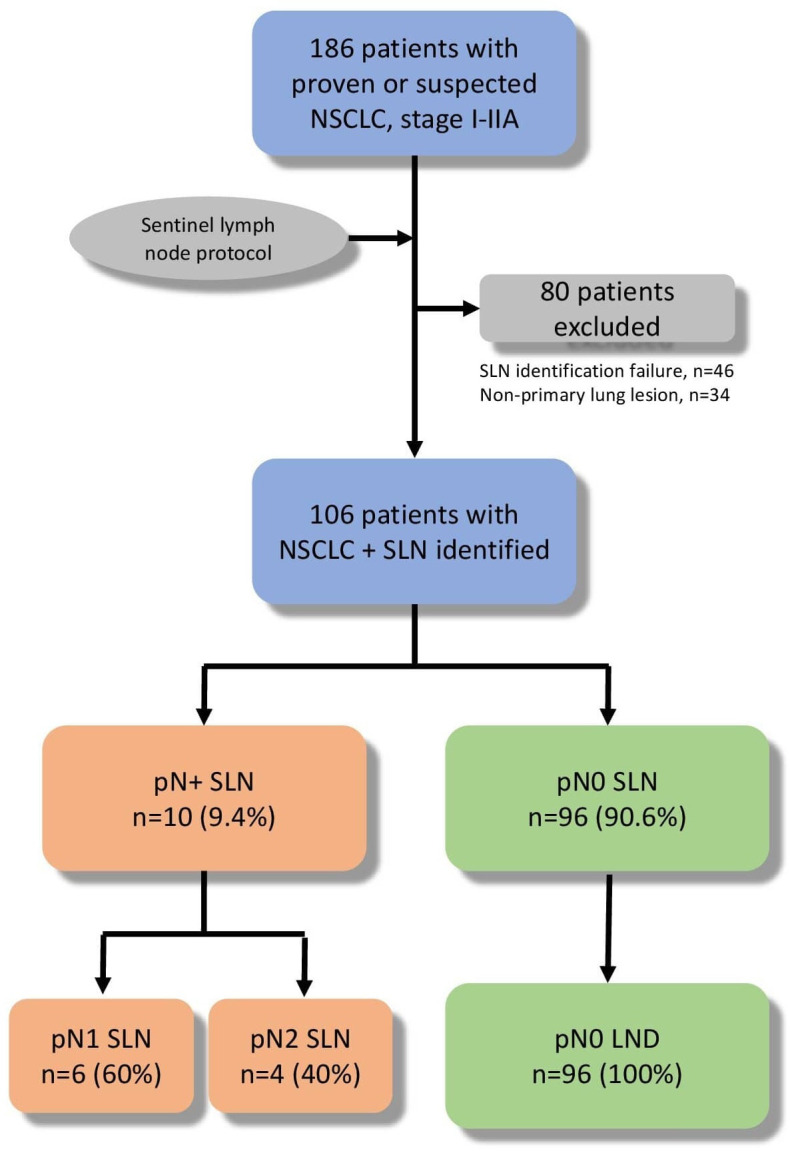
Flowchart of included patients. NSCLC, Non-Small Cell Lung Cancer; SLN, Sentinel Lymph Node; LND, Lymph node dissection.

Altogether, 186 patients were consented to undergo sentinel lymph node identification (**Figure 2**). Of those that benefitted from our SLN protocol, 80 patients were excluded from our analysis due to histological results of not being a primary lung cancer (n=34; 18.3%) and failure to identify the SLN (n=46; 24.7%). Failures to identify SLN were mostly observed at the beginning of the experience with ENB and were: equipment malfunction (n=10; 21.7%), anatomical difficulties to reach the nodule (n=25; 54.3%), ICG extravasation in the pleural cavity due to injection-related pleural effraction (n=11; 24%).

Patients were majority female (n=54; 50.9%), ex-smokers (n=63; 59.4%), and clinical stage IA2 (n=50; 47.2%; **Table 1**). All patients (n=106) were suspected of having pathologically node negative disease (N0) based on preoperative assessments (CT scan and 18F-FDG PET scan) for stage IA and on endobronchial ultrasound-guided transbronchial needle aspiration (EBUS-TBNA) for stage IB-IIA. Of the procedures performed, 55.7% (n=59) were lobectomies and 44.3% (n=47) were segmentectomies. The surgical approach was mainly minimally invasive: 82.1% by Video-Assisted Thoracoscopic Surgery (VATS) and 8.5% by Robotic-Assisted Thoracoscopic Surgery (RATS). Thoracotomy was performed in 9.4% of cases.

**Table 1:** Patient demographics.

	Study population (n=106)
**Sex, male/female**	52/54 (49.1%/50.9%)
**Age in Years (mean ± SD)**	66.7 ± 7.8
Smoking Status	
Never	19 (17.9%)
Ex-Smoker	63 (59.4%)
Current	24 (22.7%)
Type of Resection	
Lobectomy	59 (55.7%)
Segmentectomy	47 (44.3%)
Surgical Approach	
VATS	87 (82.1%)
Robotic	9 (8.5%)
Thoracotomy	10 (9.4%)
Pulmonary Function	
FEV1(mean % ± SD)	91.7 ± 19.4
DLCO (mean % ± SD)	78.7 ± 19.4
Clinical Staging	
IA1	23 (21.7%)
IA2	50 (47.2%)
IA3	18 (16.9%)
IB	13 (12.3%)
IIA	2 (1.9%)
Suspected Nodal Stage	
N0	106 (100%)
Charlson Score	5.07 ± 1.60

Patient characteristics presented with mean and standard deviation (SD). VATS, Video-Assisted Thoracoscopic Surgery; FEV1, Forced Expiratory Volume in One Second; DLCO, Diffusing Capacity of the Lungs for Carbon Monoxide.

When implementing the SLN protocol, 88 patients (83%) were injected with ICG via ENB and the remaining 18 patients (17%) were marked transpleurally. Median navigation time was 11.8 minutes (SD: 5.9) and the median SLN identification time was 6.9 minutes (SD: 5.5).

Most of our patients presented with a single station SLN (n=100), while a minority had multi-station SLN (n=6; **Table 2**). We observed in the single station cohort 16 intra-parenchymal lymph nodes and 52 lymph nodes in N1 stations. There were 32 lymph nodes in mediastinal stations, resulting in a 32% “Skip-N2” SLN rate. Of interest, we also observed SLN in stations not routinely included in SLND, particularly in station 3a. Sentinel lymph node sites relative to tumor positions (including “multistation” sentinel lymph nodes) are disclosed in **Table 3**. The average number of lymph nodes resected was 14, with a with a range from 12 to 66.

**Table 2:** Sentinel node locations.

	Single SLN (n=100)
Mediastinal Stations	
3a	1 (1%)
4	6 (6%)
5	14 (14%)
7	9 (9%)
9	2 (2%)
Hilar Stations	
10	21 (21%)
11	26 (26%)
12	5 (5%)
Intra-parenchymal nodes	16 (16%)
	Multiple SLN (n=6)
7 + 4	2 (33%)
7 + 3a	2 (33%)
7 + 10	2 (33%)
	pN+ SLN (n=10)
5	4 (40%)
10	2 (20%)
11	2 (20%)
12	1 (10%)
Intra-parenchymal nodes	1 (10%)

SLN, Sentinel Lymph Node; pN+, Positive Lymph Node.

**Table 3:** Sentinel lymph node stations relative to tumor positions (including “multistation” sentinel lymph nodes).

	RUL (n=39)	RML (n=5)	RLL (n=21)	LUL (n=36)	LLL (n=5)
**2**	0	0	0	0	0
**3a**	0	0	2	1	0
**4**	6	0	2	0	0
**5**				14	0
**7**	7	0	6	0	2
**8**	0	0	0	0	0
**9**	0	0	2	0	0
**10**	9	2	0	10	2
**11**	7	2	9	6	2
**12**	2	0	1	2	0
**Intra-parenchymal**	10	1	1	3	1

RUL, Right Upper Lobe; RML, Right Middle Lobe; RLL, Right Lower Lobe; LUL, Left Upper Lobe; LLL, Left Lower Lobe.

Of our suspected N0 patients, 96 patients (90.6%) were identified as having a non-malignant SLN, while malignant invasion was present in 10 patients (9.4%; **Table 4**). This is a 9.4% upstaging rate. All the identified negative nodules were confirmed negative by our pathology department and similarly all identified positive SLN were analyzed and deemed to be malignant by a pathologist. This gives us a 100% pathological concordance between our intraoperative SLN identification and pathological results. Of the 10 patients with a pN+ sentinel lymph node, 2 had undergone left upper lobe S1-S2-S3 segmentectomy. The cases were presented to a multidisciplinary tumor board and medical management with chemotherapy followed by surveillance was chosen.

**Table 4:** pN0 and pN+ patients’ demographics.

	pN0 patients (n=96)	pN+ patients (n=10)	p value
**Sex, male/female**	46/50 (47.9%/52.1%)	6/4 (60%/40%)	0.69
**Age in Years (mean ± SD)**	68.5 ± 10.25	63 ± 5	0.12
Smoking Status			
Never	19 (19.8%)	0 (0%)	0.25
Ex-Smoker	55 (57.3%)	8 (80%)	
Current	22 (22.9%)	2 (20%)	
Type of Resection			
Lobectomy	51 (53.1%)	8 (80%)	0.19
Segmentectomy	45 (46.9%)	2 (20%)	
Surgical Approach			
VATS	78 (81.3%)	10 (100%)	0.32
Robotic	8 (8.3%)	0 (0%)	
Thoracotomy	10 (10.4%)	0 (0%)	
Pulmonary Function			
FEV1 (mean % ± SD)	94 ± 26.25	96 ± 7	0.32
DLCO (mean % ± SD)	75.5 ± 22.75	102 ± 4	<0.01
Clinical Staging			
IA1	22 (22.9%)	0 (0%)	<0.01
IA2	47 (49%)	4 (40%)	
IA3	12 (12.5%)	6 (60%)	
IB	13 (13.5%)	0 (0%)	
IIA	2 (2.1%)	0 (0%)	
Pathological Nodal Involvement			
N0	96 (100%)	/	
N1	/	6 (60%)	
N2	/	4 (40%)	
Histopathological diagnosis			
Adenocarcinoma	66 (68.8%)	10 (100%)	0.11
Squamous cell carcinoma	23 (24%)	0 (0%)	
Carcinoid	7 (7.2%)	0 (0%)	

Patient characteristics presented with mean and standard deviation (SD). VATS, Video-Assisted Thoracoscopic Surgery; FEV1, Forced Expiratory Volume in One Second; DLCO, Diffusing Capacity of the Lungs for Carbon Monoxide.

**Figure 3:**
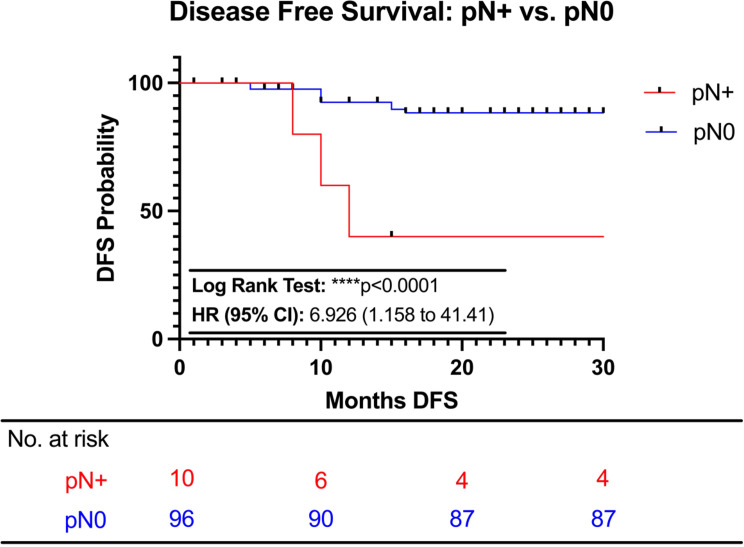
Disease free survival of sentinel lymph node positive (pN+) vs. sentinel lymph node negative (pN0) patients.

The median follow-up time for this cohort was 20 months (IQR=13.25). DFS was significantly lower in the pN+ SLN group (median of 20 months (IQR=5) than in the pN0 SLN group (median not reached) (p<0.0001; **Figure 3**). In pN1 SLN group, the median DFS is not reached, and in pN2 SLN group, the median DFS is 10 months (IQR=3). A single local recurrence (para-aortic lymph node) and 8 distant recurrences [Lung (n=2); Bone (n=2); Liver (n=2); Brain (n=2)] occurred within the pN0 sentinel group. This represents a recurrence rate of 9.4% in this group. In the pN+ group, there were 6 pN1 patients and 4 pN2 patients. In the 6 pN1 patients, the recurrence rate was 33.3%, with 2 local recurrences [4R lymph node (n=2)] and no distant recurrences. In the 4 pN2 patients, the recurrence rate was 100% with 2 local recurrences [4L lymph node (n=2)] and 2 distant recurrences (Adrenal Gland and Cerebellum [n=1); Generalized Distant Recurrence (n=1)]. OS outcome was not reached in either of the groups.

## 4 Discussion

As the largest published cohort of SLN mapping in lung cancer surgery, our study has shown a 100% concordance between the SLN and the rest of the LND. In particular, we have shown that in case of absence of SLN involvement by neoplastic cells, the remaining lymph nodes are free of cancer.

The pathological concordance between SLN and the rest of the overall LND has been poorly studied in the literature. In the few published studies that have investigated SLN, including our work, the studied population was mainly focused on lung cancer early-stages. Indeed, lung lymphatic drainage remains segmental (2) and large tumors frequently extend over several segments in the same lobe. The risk with ICG injection is that, depending on the site of the tumor injection, it may only reveal a sentinel lymph node specific to the part of the tumor which was injected. As a result, the risk of a false negative seems significantly increased. Nevertheless, concerning pathological concordance, our results are in line with previous series. So far, there are only 2 published cohorts with a very small number of patients, which found 100% concordance between SLN and the LND after surgical resection of lung cancer (22, 23). In the Kawakami et al. study, SLN biopsy using ICG was performed on 22 patients who had cT1 or T2N0M0 lung cancer. The ICG injection was only performed by transpleural injection. Their SLN identification rate was 72,7%, in line with our results for the same injection approach. Thirteen of the 16 SLNs identified were pN0 and associated with a LND free of cancer cells. Digesu et al. included 42 patients with NSCLC who underwent peritumoral injection of ICG mainly transpleurally and in few cases by ENB (23). The SLN was identified in 23 patients (54.7% identification rate, explained by an initial dose-scalation trial). Of these 23 SLNs, 16 were SLN pN0. In 100% of patients with a pN0 SLN, the rest of the LND was pathologically N0. Our study has the particularity and originality of performing the ICG injection mainly by ENB. Apart from this, the design of our study is quite similar to the other 2, with comparable results in terms of pathological concordance between the SLN and the other lymph nodes. Nevertheless, this high concordance rate should be interpreted with caution due to highly selected patients (very localized stages) and the limited number of patients in the cohorts. They also revealed that comparing SLN versus non-SLN pN0 patients, the probability of 5-year DFS is statistically significantly improved at 100% versus 66.1% (p=0.036), respectively. Disease-free survival for patients with pN+ disease appeared low in our study with a median of 20 months. This may be explained by the low number of patients in the pN+ SLN group even though a correlation between the presence of lymph node micrometastases and poor prognosis has been observed (24). Nosotti et al. shown a statistically significant differences in the disease-free intervals between patients with resected stage I NSCLC with and without micrometastases, with a median of 30 months in case of micrometastases in the intra-thoracic lymph nodes (25).

We noticed a lower SLN identification rate with the transpleural approach (72.7%) in comparison with the ENB approach (77.8%), without any significant difference. However, ENB seemed to offer a more physiological diffusion of ICG in lymphatic vessels. We observed more diffusion of ICG on the lung surface and in the chest cavity, and multiple SLN in case of direct transpleural injection, probably related to visceral pleural effraction by the needle. These may explain the preferential use of ENB (77,4%) and the better identification rate of the lymph node with this approach in our findings.

Regarding the pN0 sentinel group of our study, we noted a local recurrence at station 5 in a patient who had undergone a left S1-S2 segmentectomy for an adenocarcinoma. This observation leads us to hypothesize that this patient could have been a false-negative (false SLN pN0). Lymph node fluorescence was intraoperatively observed in station 5, and the most accessible lymph node was harvested. After clearance of this lymph node, there was still fluorescence deep under the aorta. As fluorescence can be seen up to 3 cm deep, we can consequently hypothesize that the wrong lymph node was collected and considered as the sentinel lymph node. This strengthens the fact that lymph node fluorescence should be confirmed on a table in the operating room after removal, to avoid improper labelling of the SLN.

In contrast to the management of other cancers such as breast cancer or melanoma, SLN identification does not currently play a role in the surgical standard of care for NSCLC. To draw a parallel with the role of SLN in breast cancer, axillary lymph node dissection was a procedure originally designed to maximize survival, regional control, and to determine the stage of the tumor (26). Axillary LND is associated with significant morbidity, including high rates of lymphedema, pain, dysesthesia of the upper limb and worse quality of life (27, 28). The advent of SLN techniques for stages T1-T2 N0 has made it possible to minimize these side effects (29). In addition, several studies have shown that there is no difference in terms of OS, DFS and local control between women who have or have not had additional axillary LND in case of a pN0 SLN (30). Suggesting the absence of a therapeutic role for axillary LND, complete LND has been performed less and less for localized stage cancers when SLN is negative. To go a step further, until the early 2010s, axillary LND was the standard in cases of micro or macrometastatic invasion of SLN (31). Developments in breast cancer management and the choice of systemic treatment based on the biological characteristics of the tumor have raised questions about the need for axillary LND in some patients with sentinel node metastases. The ACOSOG Z0011 randomized clinical trial showed that women with cT1-T2 N0 breast cancer and 1 or 2 positive SLN without associated additional axillary LND did not have inferior OS or DFS than those in whom additional axillary LND was performed (32). The IBCSG 23-01 study confirmed these results (33) and since then, international recommendations (American Society of Clinical Oncology, National Comprehensive Cancer Network) have not recommended additional axillary excision in cases of macro or micrometastatic invasion of SLN, if all the ACOSOG Z0011 inclusion criteria are met (34). In the era of immunotherapy and targeted therapies, practices concerning LND in breast cancer could be transposed to lung cancer.

The extent of LND for localized stages of NSCLC remains a debated topic due to the low rate of lymph node metastases in these stages and the potentially associated complications (35, 36). Even if they are rare, complications linked to LND can occur such as bleeding, recurrent laryngeal nerve damage and chylothoraxes (37).

Several animal model studies have shown that the primary sites for the generation of tumor-specific T lymphocytes are the lymph nodes draining the tumor (38). A study in 2021 using a murine model showed that mice operated on for micrometastatic lung cancer without associated LND had better survival than mice in which LND was carried out (39). This suggests that LND may affect the anti-tumor immune response. However, an even more recent study in humans showed that extensive lymph node dissection (number of resected lymph nodes ≥ 16) was associated with a reduction in the efficacy of immunotherapy in the case of recurrence after surgical resection (40). This phenomenon is understood to be due to an alteration in anti-tumor immunity, calling for the need of a immune-focused LND dissection strategy that emphasizes preservation of the immune system, i.e. a non-extensive lymphadenectomy that will enable quality staging to be performed while avoiding damage to the immune system (40). This is where SLN identification, and its surrogate association to overall LND pathology, could come into importance for therapeutic regimens.

Recently, some studies have examined the outcomes of adjuvant immunotherapy after upfront surgical resection. The IMpower-010 study evaluated the benefits of adjuvant immunotherapy for 1 year after initial surgical resection of NSCLC, followed by adjuvant chemotherapy (41). It demonstrated that atezolizumab significantly improved DFS in patients with stage II-IIIa NSCLC with Programmed death-ligand 1 (PDL1) expression greater than or equal to 1%, when compared to patients who did not receive anti-PDL1 treatment. Notably, atezolizumab is not available in many regions of Europe with the same criteria. For example, the reimbursement of costs has been refused by the French Haute Autorité de Santé in January 2023. Nevertheless, it is debated whether there is any real benefit in performing extensive LND in early-stage NSCLC, which could dampen the immune system, in the adjuvant setting.

Taken together, these innovations and considerations question the role of extensive LND, as it may impede the efficacy of adjuvant regimens. We acknowledge that LND does remain the standard for advanced stage patients or for patients who have been treated with a neoadjuvant therapy, as complete LND makes it possible to assess the tumors’ pathological response and determine a patient’s nodal downstaging (42). Nevertheless, it is not well understood whether there is any real benefit in performing extensive LND in localized NSCLC, with a goal of administering an adjuvant therapy after upfront surgical resection (43). On a cellular level, cells such as central memory T cells (T_CM_) are involved in anti-tumor immunity and are naturally present in draining lymph nodes. Hence aggressive lymphadenectomy could impair this mechanism of immunosurveillance, aiding tumor progression (44).

Systematic lymph node dissection is the gold standard for NSCLC surgery, however lobe-specific LND remains a possible alternative especially for localized stages (45). In our series, the SLN was in most of the cases in the drainage area of the lobe, so a lobe-specific dissection may have harvested it. On the other hand, we observed in 3 cases, atypical location of the sentinel lymph node (in the 3A station), which is usually not dissected by most teams during the surgery. Even though the lobe-specific LND might allow to harvest in the large majority of cases the SLN, it might still remove lymph nodes implied in the anti-tumor immunity. Moreover, all of the pN+ SLN had a micro-metastatic involvement, which was not detected by the usual analysis but revealed by immune-histochemistry analysis. One can wonder whether in case of lobe-specific LND immuno-histochemistry analysis could be performed on all harvested lymph nodes. However, this may lead to increased pathological examination costs and delay in pathological reports. Therefore, in patients with a pN0 SLN, the advantages of avoid SLND or lobe-specific LND are numerous. Firstly, quality staging can be achieved while minimizing the risks of LND (bleeding, chylothorax, nerve damage). This may therefore potentially reduce the length and cost of hospital stay. Secondly, the anti-cancer immune system is preserved, as is its ability to reactivate in the event of tumour recurrence.

Our study has certain limitations that need to be considered: it is a single-center experiment with a small population, despite being the largest SLN cohort in the literature. The SLN technique, by ENB, carries a certain cost that needs to be covered. On the other hand, the cost of ENB could be balanced by direct transpleural injection. Indeed, we did not observe any significant difference between the 2 injection techniques in terms of SLN identification rate (p=0.86). However, we noticed more extravasation of ICG into the chest cavity and multiple SLN with transpleural injection. Hence, this approach seems to be less physiological and accurate compared to ENB. Anaphylactic reactions have been reported in the literature, but we did not observe any in our study. As far as overall survival is concerned, we do not currently have sufficient data to make a definitive statement. With time and greater generalization of this technique, as a community, we will be able to assess this outcome with confidence. Finally, the technique and failures in identifying the SLN are notable. It comes with great difficulty to assess why we experienced an inability to identify the SLN in the few excluded patients. There is a possibility that the SLN was in a more distal anatomic location that is not traditionally dissected. This greater distance might within itself be a limitation of the depth at which the infrared camera can detect a signal. Another possibility is that the injection was not properly placed. While these are a limitation of the technique, it does not inhibit the concordance analysis of this study.

Having already shown that the SLN technique is feasible in thoracic surgery using ICG and NIR-imaging, we have now demonstrated a 100% pathological concordance between when the SLN is cancer-free and the rest of the lymph nodes in the LND. With the development of screening programs enabling more and more early-stage lung lesions to be diagnosed (46), and the gradual integration of immunotherapies in the adjuvant setting, these data could change future practices concerning LND. Indeed, this SLN technique appears to be a key element for identification of the true first lymph node relay (47), increased detection of micro metastases (48) and preservation of the local immune system (49). However, more studies involving larger populations and longer follow up are needed to confirm these results and clarify the role of SLN in the management of early-stage lung cancer.

## Data Availability

The original contributions presented in the study are included in the article/supplementary material. Further inquiries can be directed to the corresponding author.
